# 4-Chloropropofol enhances chloride currents in human hyperekplexic and artificial mutated glycine receptors

**DOI:** 10.1186/1471-2377-12-104

**Published:** 2012-09-24

**Authors:** Jeanne de la Roche, Martin Leuwer, Klaus Krampfl, Gertrud Haeseler, Reinhard Dengler, Vanessa Buchholz, Jörg Ahrens

**Affiliations:** 1 Clinic for Anesthesia and Critical Care Medicine, OE 8050, Hannover Medical School, Carl-Neuberg-Str. 1, 30625, Hannover, Germany; 2 Critical Care Research Unit, The University of Liverpool, Daulby Street, Liverpool, L69 3, GA, U.K; 3 Department of Neurology and Neurophysiology; OE 7210, Hannover Medical School, Carl-Neuberg-Str. 1, 30625, Hannover, Germany; 4 Clinic for Anesthesia and Critical Care Medicine, St. Elisabeth-Hospital Dorsten, Pfarrer-Wilhelm-Schmitz Str., 1 46282, Dorsten, Germany

**Keywords:** Glycine receptor mutations, Hereditary hyperekplexia, 4-chloropropofol

## Abstract

**Background:**

The mammalian neurological disorder hereditary hyperekplexia can be attributed to various mutations of strychnine sensitive glycine receptors. The clinical symptoms of “startle disease” predominantly occur in the newborn leading to convulsive hypertonia and an exaggerated startle response to unexpected mild stimuli. Amongst others, point mutations R271Q and R271L in the α_1_-subunit of strychnine sensitive glycine receptors show reduced glycine sensitivity and cause the clinical symptoms of hyperekplexia.

Halogenation has been shown to be a crucial structural determinant for the potency of a phenolic compound to positively modulate glycine receptor function.

The aim of this *in vitro* study was to characterize the effects of 4-chloropropofol (4-chloro-2,6-dimethylphenol) at four glycine receptor mutations.

**Methods:**

Glycine receptor subunits were expressed in HEK 293 cells and experiments were performed using the whole-cell patch-clamp technique.

**Results:**

4-chloropropofol exerted a positive allosteric modulatory effect in a low sub-nanomolar concentration range at the wild type receptor (EC_50_ value of 0.08 ± 0.02 nM) and in a micromolar concentration range at the mutations (1.3 ± 0.6 μM, 0.1 ± 0.2 μM, 6.0 ± 2.3 μM and 55 ± 28 μM for R271Q, L, K and S267I, respectively).

**Conclusions:**

4-chloropropofol might be an effective compound for the activation of mutated glycine receptors *in experimental models of startle disease*.

## Background

Hereditary hyperekplexia also known as ‘startle disease’, ‘Kok disease’ or ‘stiff baby syndrome’ is a rare hereditary neurological disorder which is caused by mutations in genes encoding proteins involved in glycinergic neurotransmission, including the α_1_-subunit of the strychnine sensitive glycine receptor (GlyR) [1-3]. It predominantly manifests in the newborn with an extreme exaggerated hyperexcitability in terms of an abnormal startle response to sudden, unexpected acoustic, visual or somatosensory stimuli. Patients exhibit an intense tremor of arms and legs. Frequent falling attacks with episodes of convulsive hypertonia occur in adult patients [4,5]. In addition to mutations in the genes GLRB (encodes glycine receptor β-subunit), SLC6A5 (encodes glycine transporter 2) and GPHN (encodes the integral membrane protein gephyrin), mutations in the gene GLRA1 (encodes the α_1_-subunit of the GlyR) account for 40 - 80% of hyperekplexia [6,7]. The most common mutations reported are R271L or R271Q [8].

Fast inhibitory postsynaptic transmission in the central nervous system (CNS) is mainly mediated by γ-aminobutyric acid_A_ (GABA_A_) receptors, whereas glycine receptors play a major role in the spinal cord, brainstem and retina [9]. The GlyR mutations α_1_R271Q- and α_1_R271L are commonly associated with clinically relevant symptoms in patients with autosomal dominant hyperekplexia, characterized by an impaired GlyR-function due to reduced glycine sensitivity [7,10-12]. The artificial mutation R271K shows the same startle GlyR features [13,14]. S267 mutations also display startle symptoms resulting from structural alterations of the GlyR. The corresponding mutated genes have been found in hyperekplexic patients and animals [15,16]. Patients suffering from startle disease are commonly treated with GABA_A_-activating drugs like clonazepam [12]. Clonazepam relieves the symptoms of hyperekplexia indirectly, but may be accompanied by sedative side effects [17].

**Figure 1:**
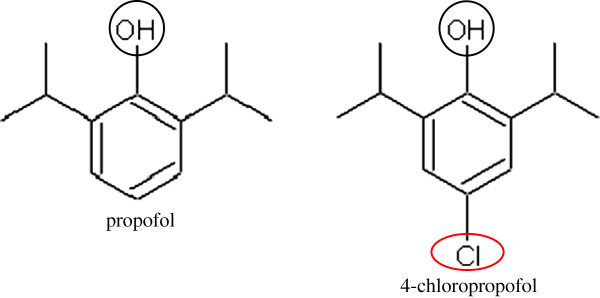
**Chemical structures of 4-chloropropofol and the anesthetic propofol.** Highlighted structural features are the non-substituted phenolic hydroxyl group (circle) with the chloride (red ellipse) in *para*-position to the hydroxyl group.

As there is a compelling connection between startle disease and a distinct GlyR-malfunction, it would be of benefit to discover selective positive allosteric GlyR-modulators which might attenuate the hyperekplexic symptoms by restoring the function of the GlyR. Thus, it is of interest to modify the molecular structure of propofol in order to optimize all its various (aesthetic, sedative, anticonvulsant) activities or to yield drugs with more selective actions. The intravenous aesthetic propofol, well known for its positive allosteric modulatory effects at GABA_A_-receptors, has been shown to modulate glycine receptors in rat cortical and murine spinal neurons as well as in recombinant expression systems [18-21]. The effects exhibit a non-selective manner, i.e. the effects at GlyRs require higher concentrations than the effect at GABA_A_-receptors [19]. It has previously been shown that halogenation of a propofol analogue did not increase GABA-ergic activity [22,23]. A study of our group on heterologously expressed α_1_β glycine receptors found that 4-chloropropofol is almost 1000-fold more potent than propofol in enhancing glycine induced currents at wild-type (WT) glycine receptors [24]. The chemical structures of propofol and its analogue 4-chloropropofol are illustrated in Figure 1. 

The aim of this study was to investigate whether 4-chloropropofol improves the function of glycine receptor mutations relevant for the generation of hyperekplexia. Consequently, we investigated the effects of 4-chloropropofol at WT glycine receptors and at the glycine receptor mutations α_1_R271Q-, α_1_R271L-, α_1_R271K and α_1_S267I.

## Methods

### Cell culture, transfection

Human α_1_-, α_1_R271Q-, α_1_R271L-, α_1_R271K and α_1_S267I-GlyR subunits were transiently transfected into human embryonic kidney cells (HEK 293, ATCC, Manassas, USA). The wild type α_1_-GlyR -cDNA was cloned in pCIS2 (Invitrogen, San Diego, USA) vector and provided by Prof. Heinrich Betz (Max-Planck-Institut für Hirnforschung, Frankfurt am Main, Germany) [25]. For the plasmid cDNA of the mutated α_1_R271Q-, α_1_R271L-, α_1_R271K- and α_1_S267I-GlyR the eukaryotic expression vector pcDNA1amp (Invitrogen, San Diego, USA), under the control of cytomegalovirus promoter, was used. For site-directed mutagenesis, single stranded template cDNA was synthesized from M13 origin of replication and the mutations of arginine residue (R) at position 271 to glutamine (R271Q), leucine (R271L) or lysine (R271K) and mutation of serine residue (S) at position 267 to isoleucine (S267I) were generated using standard procedures [26]. The fidelity of the mutagenesis reaction was confirmed by standard didesoxynucleotide sequencing (fmol DNA Sequencing System Promega, Southhampton, UK) and mutated GlyR-cDNA was provided by Jeremy J. Lambert (Ninewells Hospital and medical school, Dundee). Wild type and mutated α_1_-GlyR subunits efficiently form homomeric receptors in heterologous expression systems [13,25,27].

HEK 293 cells were cultured in medium containing HAMS’F-12 (Biochrom, Berlin, Germany), supplemented with 10% fetal bovine serum (FBS, Biochrom, Berlin, Germany), 100 U ml^–1^ penicillin and 100μgml^–1^ streptomycin (Gibco BRL, Life Technologies, Karlsruhe, Germany) at 37°C in a 5% CO_2_/ 95% air incubator. For transfection cells were suspended in a buffer containing 50mM K_2_HPO_4_ (Fluka BioChemika, Seelze, Germany), 20mMK-acetate (Sigma-Aldrich, Taufkirchen, Germany) and 25mM MgSO_4_ (Sigma-Aldrich, Taufkirchen, Germany) at pH 7.35. To visualize transfected cells, they were co-transfected with cDNA encoding for enhanced green fluorescent protein (EGFP) contained in the pEGFP-N1 expression vector (Clontech, Palo Alto, USA). The corresponding cDNA (5μg for the GlyR and 2.5μg for EGFP) was added to 400μl of the cell suspension and the mixture was rapidly transferred into the electroporation cuvette. For transfection we used an electroporation device by EquiBio (Kent, UK) and a 4mm aluminum electrode electroporation cuvette (Peqlab, Erlangen, Germany).

Transfected cells were replated on 12mm glass coverslips (Karl Hecht KG, Sondheim, Germany) in a 24-well-plate filled with medium and incubated 15–24h before recording.

### Solutions

The phenol derivative 4-chloropropofol (2,6-dimethyl-4-chlorophenol) was provided as pure substance by Prof. Paul M. O’Neill (University of Liverpool, England), prepared as light-protected 1M stock solution in ethanol (EtOH, J.T.Baker, Griesheim, Germany) and stored in glass vessels at −20°C. The stock solution was directly dissolved in a low concentrated glycine solution (EC_20_ – positive allosteric modulation) or bath solution (direct activation) to reach the final drug concentration of 1000μM 4-chloropropofol. The investigated concentrations (0.015 nM −100μM) were calculated from the amount injected into the glass vials. Glycine (Sigma-Aldrich, Taufkirchen, Germany), 3μM −300mM, was dissolved directly into the bath solution.

Drug-containing vials were vigorously vortexed for 30min. Patch electrodes were filled with an intracellular solution of [mM] KCl 140, MgCl_2_ 2, EGTA 11, HEPES 10, glucose 11, CaCl_2_ 1 with pH 7.3, adjusted with 1 M KOH and a bath solution contained [mM] NaCl 162, KCl 5.31, NaHPO_4_ 0.85, KH_2_PO_4_ 0.22, HEPES 15, glucose 6.11, pH 7.4 adjusted with 1 M NaOH. Osmolarity of both solutions was set at 280–300 mOsmol.

It has previously been shown that osmotic controls up to 500mM sucrose produced no currents [13,27]. 300 mM glycine adjusted to pH 7.4 by Na-OH revealed 1096mOsm. High glycine solutions of 300 mM glycine osmolarity subtracted from the osmolarity of the buffer solution itself, resulted in a total of Δ 800 mOsmol. We excluded possible osmotic effects of high concentrations of glycine (up to 300 mM in our experiments) by performing experiments with 1100mM sucrose. These experiments showed a lack of osmotic effects on HEK 293 cells transfected with WT glycine receptors [see Additional file 1. Bath solution itself did not induce any current amplitude. Thus, any direct effects of 4-chloropropofol in the absence of glycine can be directly attributed to the applied substance.

### Experimental set-up

Whole-cell experiments [28] were performed at a holding potential of −30 mV with a mean seal resistance of 1 GΩ. Chloride inward currents, due to agonist-induced channel activation, were resolved in the pA range. A fast liquid filament switch technique was used for the application of the agonist, presented in pulses of 2 s duration every 20 s. The liquid filament switch technique is able to exchange the solution passing an outside-out patch or small whole-cells within 1–2 ms [29,30]. We calculated flow rate of background solution through the chamber with 3,4 ml/min corresponding to 4% of maximal pump rate. With regard to the chamber volume of 100 μl the exchange solution time within the chamber was assessed to approximately 1.8 ms, dependent on cell size accordingly. Piezo-switch and small diameter capillary (ID 0.15 mm) within the measurement chamber promoted the millisecond solution exchange. The correct positioning of the cell, in respect to the liquid filament (ID 0.15 mm), was ensured by applying a saturating glycine pulse (1 mM for WT and S267I, 300 mM for R271Q and L, 10 mM for R271K) before and after each test experiment [see Additional file 2. The induced current (I) by this saturating control solution was defined as I_control_. Care was taken that the amplitude and shape of the glycine induced control currents had stabilized before proceeding with the experiment. The stability of the seal was controlled during the complete experiment *via* control of the seal resistance. A variation of 10-20% of the basic value was regarded as tolerable. Test solution and the saturating glycine solution were applied *via* the same glass-polytetrafluoroethylene perfusion system, but from separate reservoirs.

4-chloropropofol was applied either alone, in order to determine its direct agonistic effects at the GlyR mutations or in combination with a sub-saturating (EC_20_) glycine concentration (20 μM for WT, 10 mM for R271Q, 30 mM for R271L, 100 μM for R271K and 30 μM for S267I), in order to determine its glycine modulatory effects. A new cell was used for each protocol and at least four different experiments were performed for each condition. The concentration of the diluent EtOH corresponding to the highest drug concentration used was 17150 μM. We have performed experiments demonstrating the lack of effect of ethanol on the potentiation of glycine induced currents in this concentration [see Additional file 3].

### Current recording and analysis

For data acquisition we used an EPC 10 digitally-controlled amplifier in combination with Patch Master Software (HEKA Electronics, Lambrecht, Germany). Currents were filtered at 2 kHz. Analysis was performed using Fit Master (HEKA Electronics, Lambrecht, Germany) and Graph Prism 5.0 software (GraphPad, La Jolla, USA). Fitting procedures were performed using a non-linear least-squares Marquardt-Levenberg algorithm.

The concentration-response-curves for receptor activation by the natural agonist and for positive allosteric modulation by 4-chloropropofol were fitted according to the Hill function (I_norm_ = [1 + (EC_50_/[C])^nH^ ]^–1^). I_norm_ is the current induced by the respective concentration [C] of 4-chloropropofol in the chloropropofol-glycine mixture. EC_50_ is the concentration required to evoke a response amounting to 50% of their own maximal response and n_H_ is the Hill coefficient. For 4-chloropropofol, the dose–response curves did not always reach a plateau response, because 4-chloropropofol in high concentrations leads to a decline in seal resistance and thus, did not yield reliable results. Therefore, further curve process could not be described by the Hill function. In these cases, the maximum response was the response at the highest concentration of the test compound for which a reliable response could be recorded.

KS normality test showed no normal distribution for the calculated EC_50_ values; consequently two-tailed Mann–Whitney U test was performed to determine significance. All columns depicted in the diagram are means ± SEM and the levels of significance are indicated as *p < 0.05 and **p < 0.01.

Positive allosteric modulation of 4-chloropropofol was expressed as percentage of the current elicited by the sub-saturating glycine solution according to E (%) = 100 [(I-I_0_)/I_0_, where I_0_ is the current response to the sub-saturating glycine solution. Currents were normalized to their own maximum response. The rise time τ (ms) of the initial glycine current traces (10-90% of the maximal amplitude I_max_) were determined with a monoexponential fit (Fitmaster, HEKA Electronics) [31]. Data in tables and figures as well in the following results section are shown as mean values ± SEM.

A total of 90 cells was included in the study for the investigation of glycine sensitivity (n = 42) and modulating effects (n = 48) of mutant and WT glycine receptors. Cells that did not show a stable seal till the end of the experiment were excluded for EC_50_ calculation. Thus, the cells included in the calculation of I_max_ and rise time values of the peak amplitude may differ in the total number. Details, e.g. about the number of cells used for each test experiment, are provided in the appropriate figures and tables.

## Results

In our study we were able to characterize glycine sensitivity and modulating effects (positive allosteric modulation and direct activation) of α_1_ glycine receptors and its mutations R271Q, L, K and S267I by 4-chloropropofol.

### Glycine sensitivity

**Figure 2:**
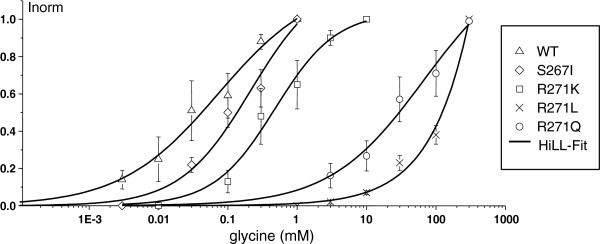
**Dose–response curves for receptor activation by the natural agonist glycine. Solid lines are Hill fits to the data (mean ± SEM) with the indicated parameters.** The EC_50_ value defines the effect at half-maximal activation and n_H_ represents the corresponding Hill coefficient.

Glycine induced larger inward currents in HEK293-cells expressing WT glycine receptors than the startle disease mutations α_1_R271Q, α_1_R271L and α_1_R271K, following application of a saturating concentration of the natural agonist. Consequently, the glycine sensitivity of the mutations to the natural agonist glycine was considerably reduced [see Additional file 4]. Glycine induced inward currents at S267I are only marginally reduced in amplitude compared to the WT. The current transient at the WT showed a biphasic time course with a fast increase, followed by a monophasic decay. These characteristics were considerably changed at the startle receptors, whereas R271K reflected to a greater extent the WT. S267I shows nearly similar rise time values compared to the WT. Concentration-response curves are depicted in Figure 2. EC_50_ values and corresponding Hill coefficients (n_H_), as well as rise time values and mean maximal current amplitudes are shown in Table 1.

**Table 1:** Glycine sensitivity at the WT and mutated glycine receptors

**glycine**	**EC**_**50**_**[mM] ± SEM**	**n**_**H**_**± SEM**	**N=**	**Mean current amplitude [pA] ± SEM**	**Rise time [ms] ± SEM**	**Current amplitude [% of mean WT current amplitude] ± SEM**
**WT**	0.04±0.02	0.8±0.7	7	1645±426	3.7±0.8	100.0±26
**R271Q**	26.3±17.7	1.0±0.6	6	123±19	673±224	7.5±15
**R271L**	102.1±15.0	1.8±0.7	5	336±51	998±129	20.5±12
**R271K**	0.43±0.29	1.0±0.5	6	526±97	14.9±7.7	32.0±18
**S267I**	0.12±0.05	1.1±0.2	4	1122±765	6.6±2.5	68.2±68

EC_50_ values, corresponding Hill coefficients (n_H_) as well as rise time values and mean maximal current amplitudes are depicted for glycine receptor activation by the natural agonist glycine.

### Positive allosteric modulation and direct-activating effects of 4-chloropropofol

4-chloropropofol potentiated the response of a sub-saturating glycine solution (EC_20_) in a concentration dependent manner resulting in EC_50_ values in the low sub-nanomolar concentration range at the wild type, in the low-micromolar to sub-micromolar concentration range at the startle mutations and in the micromolar concentration range at S267I.

**Figure 3:**
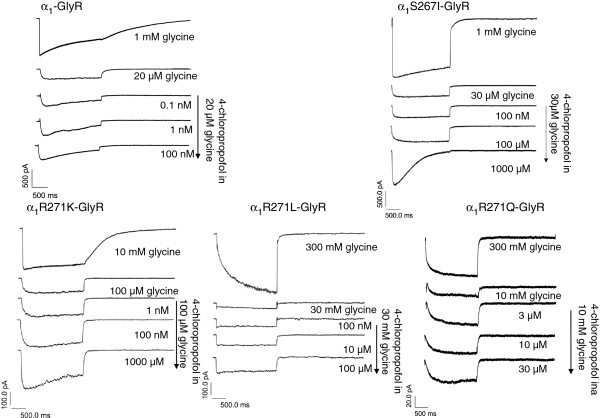
**Representative current traces for positive allosteric modulation of glycine induced currents by 4-chloropropofol at WT and mutatedα**_**1**_**-glycine receptors.** Traces elicited by a 2 s co-application of a sub-saturating glycine solution (EC_20_ = 10 μM WT, 30 μM S267I, 10 mM R271Q, 30 mM R271L and 100 μM R271K) and 4-chloropropofol. Respective upper traces show the maximal current elicited by a 1 mM (WT and S267I), 300 mM (R271Q and R271L) and 10 mM (R271K) respectively, glycine control solution. 4-chloropropofol increased the amplitude of the response evoked by glycine (second trace from top) in a concentration dependent manner (third and following traces from top).

**Figure 4:**
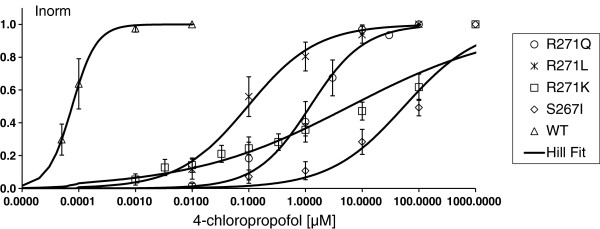
**Dose–response curves for positive allosteric modulation of glycine induced currents by 4-chloropropofol.** Solid lines are Hill fits to the data (mean **±** SEM) with the indicated parameters. The EC_50_ value defines the effect at half-maximal activation and n_H_ represents the corresponding Hill coefficient.

Concentration-response curves, representative current traces, EC_50_ values and corresponding Hill coefficients (n_H_), as well as maximal positive allosteric modulation are depicted in Figures 3, 4 and Table 2.

**Table 2:** Positive allosteric modulation of 4-chloropropofol at GlyR/mutations

**4-chloropropofol**	**positive allosteric modulation**			
	**EC**_**50**_**± SEM**	**n**_**H**_**± SEM**	**N=**	**potentiation [% of peak current amplitude] ± SEM**
**WT**	0.08 ± 0.02 nM	2.0 ± 2.8	4	269 ± 88
**R271Q**	1.3 ± 0.6μM	0.9 ± 1.3	5	260 ± 52
**R271L**	0.1 ± 0.2μM	0.7 ± 0.3	6	153 ± 46
**R271K**	6.0 ± 2.3μM	0.3 ± 0.1	6	231 ± 174
**S267I**	55.1 ± 28.4μM	0.7 ± 0.3	4	128 ± 14

EC_50_ values and Hill coefficients for receptor modulation by 4-chloropropofol derived from fits of the Hill equation to the normalized current response as well as values for maximal positive allosteric modulation at WT and mutated glycine receptors.

**Figure 5:**
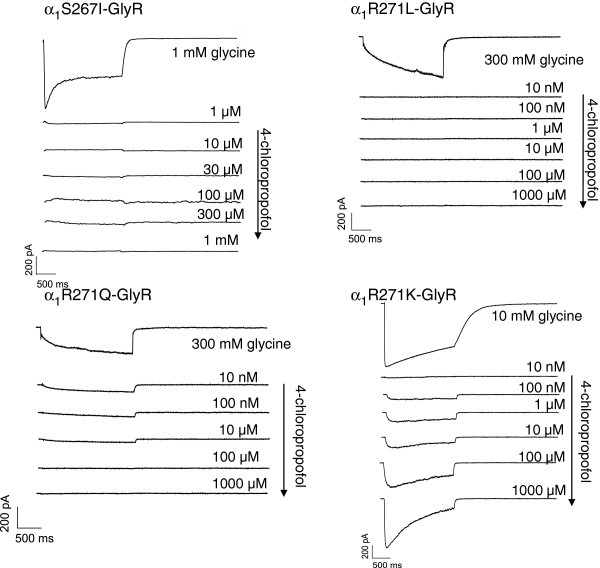
**Representative current traces for direct activation of 4-chloropropofol at startle receptors in the absence of natural agonist glycine.** Representative current traces are depicted as follows: first trace saturating glycine control and subsequent traces different doses of 4-chloropropofol.

Direct activation of the GlyR mutations by 4-chloropropofol (0.01-1000 μM) in the absence of the natural agonist glycine is inconsistent. At R271K we found a dose-dependent current increase (EC_50_ = 16.4 ± 22.3 μM, n_H_ 0.41 ± 0.15, n = 5) with a mean maximal activation of 77.2 ± 4.2%. At R271Q there was only a marginal direct activation, while R271L and S267I were completely insensitive to the direct effects of 4-chloropropofol (Figure 5).

**Figure 6:**
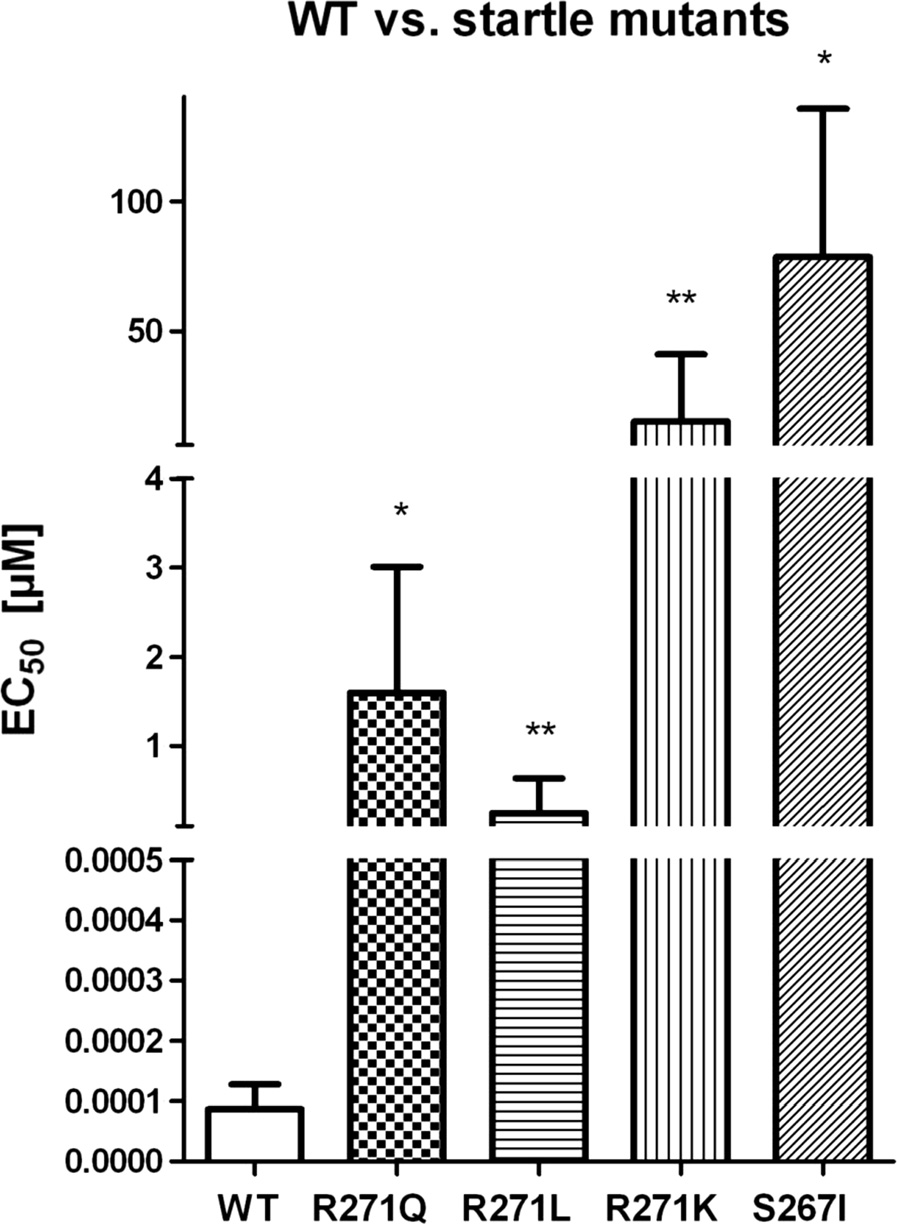
**Mean-standard deviation plot of the calculated EC**_**50**_** values (mean ± SD) at either α1-(WT) or mutations -R271Q, -R271L, -R271K and -S267I expressed in HEK293 cells following application of 4-chloropropofol.** P-values of Mann–Whitney U test result in significant differences between EC_50_ of WT and the corresponding GlyR-mutation, indicated as significance levels of *p<0.05 and **p<0.01 (WT vs. R271Q, p=0.0159; WT vs. R271L as well as vs. R271K, p = 0.0095 and WT vs. S267I, p = 0.0286; n = 4–6 per group).

The significant differences of calculated EC_50_ values for positive allosteric modulation of 4-chloropropofol at the four startle mutations and the WT are shown in a mean-standard deviation-plot in Figure 6.

## Discussion

This study provides evidence that the *para*-substituted propofol derivative 4-chloropropofol effectively modulates the WT GlyR and the α_1_R271Q, α_1_R271L- α_1_R271K- and S267I-GlyR startle mutations *in vitro*. 4-chloropropofol appears to be much more potent than propofol at the WT GlyR and at the mutation S267I [21].

These findings show a potential role for halogenated propofol derivatives in restoring the reduced function of mutated glycine receptors, which might alleviate the symptoms of the reduced glycinergic inhibition in experimental models of hyperekplexia. In a previous study we have shown that the halogenation of phenol derivatives is crucial for effective, positive allosteric modulation of the GlyR [32]. Thus, it was our aim to find a substance able to enhance the reduced glycinergic inhibition by pharmacological modulation of mutated receptors.

### Positive allosteric modulation of WT and mutated GlyR with impaired glycine sensitivity by 4-chloropropofol

We were able to demonstrate the strongly reduced glycine sensitivity of the mutated receptors associated with lower maximal inducible current amplitudes and higher EC_50_ values for glycine compared to the WT [8,13,27]. Hill slope values for alpha1 glycine receptors in HEK293 are known to show cooperativity and described to amount nH 2.4 for human [33]and nH 3.3 for rat alpha 1 GlyR [34]. The remarkably reduced nH values of our study (nH 0.8 for WT) may be due to differences in drug application speed and set up mode. The prolonged rise time of 3.7 ms for WT (vs. 0.4 ms on cell mode, 4 s drug application [35] also corroborates this hypothesis. Nevertheless, the elongated rise time values of hyperekplexic glycine receptors compared to the WT are appreciable. Our results clarify the importance of amino acid position R271 in the transmembrane domain 2 (TM2) of the α_1_ subunit for the allosteric modulation of the GlyR [36]. R271 determines the channel properties for chloride binding and entry [11,12] and is thought to be involved in channel gating [37] and ion permeation [38]. The mutations at R271 lead to a reduced chloride conductivity at mutated startle receptors whereas the influence on assembly and oligomerisation of the GlyR is reported to be negligible [13]. Charge reversal mutations of positive charged amino acids in the TM2 of the α1 subunit have been published to reduce single channel conductance by 41% [39].

4-chloropropofol effectively potentiated glycine-induced currents at the mutations R271Q, R271L and R271K. Despite the strongly reduced glycine sensitivity of the hyperekplexic receptors, modulation of receptor function was detected in the low-micromolar and sub-micromolar concentration range. Furthermore, our study shows that halogenation of the anesthetic propofol yields a compound with a highly increased potency for activation of chloride currents via WT and mutated α_1_ glycine receptors. 4-chloropropofol shows a more than 1000-fold increased potency to positively modulate glycine induced chloride currents at WT glycine receptors in comparison to the effect of propofol [21].

A recent study provided evidence that hyperekplexic R271L glycine receptors did not significantly change propofol binding and its strychnine cooperativity in comparison to the WT [40]. Our experiments revealed highly increased EC_50_ values for modulation of the mutated receptors R271Q, R271L and R271K-GlyR by 4-chloropropofol compared to the effect at the WT.

### S267 seems to be crucial for 4-chloropropofol binding pocket

Point mutations in the α_1_ subunit at the amino acid position Arg271, mainly R271Q- and R271L-GlyR, are mutations leading to clinical symptoms of the impaired GlyR function in startle disease [7]. The arginine (R) at position 271 of the α_1_GlyR is substituted by glutamine (Q) or leucine (L), respectively. The substitution of arginine to lysine (K) leads to the artificial mutation R271K, which also shows the typical startle GlyR features [13,14,27]. Mutation of the serine residue at position 267 is relevant for hyperekplexia as well and an exchange to isoleucine (S267I) diminishes binding of the anesthetic propofol [10,39]. S267 has previously been shown to be crucial for binding of propofol, ethanol and halogenated anesthetics [21,41,42]. Our results show, that S267 may be a part of the binding pocket cavity for 4-chloropropofol at the glycine receptor.

Compared to propofol [27], the increase in efficacy of 4-chloropropofol at hyperekplexic receptors in this study may be explained due to an interaction at R271 with the halogen (via a possible ion dipole interaction), additive to the already supposed binding site of propofol through the intact S267 (hydrogen bond). Further, more detailed structure-binding analysis will be necessary to substantiate these interactions. Evidence for the importance of S267 mutations in generation of hyperekplexia comes from a study showing that α_1_S267Q-GlyR knock-in mice displayed a hyperekplexic phenotype and were shown to disrupt normal GlyR function [16]. Recently, it has been found in a hyperekplexic family that a S267N point mutation influences agonist responses and ethanol modulation of the mutated receptor [15].

The nature of the TM2 residue (267) of the glycine α_1_ subunit influences the glycine modulatory effect of propofol and direct activation of the receptor by this anesthetic [21]. A comparison of the impact of such mutations on the interaction of 4-chloropropofol with glycine and GABA_A_ receptors should permit a better understanding of the molecular determinants of action of 4-chloropropofol on these structurally related receptors and may aid the development of selective modulators of mutated startle disease receptors.

These *in vitro* findings are based on homomeric human α_1_-glycine receptors. Glycinergic-neurotransmission in adults is mainly based on heteromeric α_1_β-receptors [43]. The GlyR is a ligand gated ion-channel composed of five subunits [44], comprising 2α and 3β subunits [45]. Four different α isoforms exist (α1-α4) which show a developmental and regional dependant distribution [46]. Co-expression of the α subunit has an impact on the effect of various agonists (among them the natural agonist glycine), whereas its influence on the effects of strychnine seems to be negligible [45]. Other studies revealed that the expression of homomeric α_1_ subunits in mammalian cells or *Xenopus laevis* oocytes is sufficient to generate functional receptors with pharmacological properties that are typical for the native GlyR in the spinal cord [47,48]. We have previously shown that co-expression of the glycine β subunit does not affect the response of heterologously expressed WT α_1_ subunits to different halogenated phenol derivatives and propofol [21,32]. Additionally, studies with startle disease transgenic mice showed only a small level (25% of normal) of β subunits to be necessary for a proper GlyR-function [49].

Recessive GLRA1 mutations as being caused by nonsense or frameshift mutations in the α1 gene have been increasingly associated with case reports elucidating the molecular genetics of hyperekplexia [6] As autosomal dominant (AD) startle mutations of GLRA1 such as missense mutations R271Q and R271L define case reports with high frequency in hyperekplexic patients, it is important to mention that these AD cases represent only 5 of 30 index cases as recently published [6]. Cell surface expression and Imax of recessive GLRA1 mutations are markedly reduced compared to AD mutations. It would be an interesting attempt in functional analysis of 4-chloropropofol to investigate the effects of 4-chloro-PRO at recessive startle mutations.

### In vivo effects of 4-chloropropofol in experimental models of startle disease

The impact of glycine receptor modulation by propofol on the clinical effects remains an interesting, yet unresolved issue. Evidence that an effect on glycine receptors may occur *in vivo* comes from other studies of the startle disease. The intravenous anesthetic propofol is known to activate glycine-induced currents in startle mutations *in vitro* and emerged as a transient treatment of startle symptoms in a transgenic mouse line carrying the R217Q mutation (tg271Q-300). Patch-clamp experiments at different startle mutations expressed in *Xenopus laevis* oocytes revealed that propofol in the concentration range of 1–500 μM increases the glycine induced maximal response. Transgenic mice (tg271Q-300) exhibited a decreased righting time and abatements in tremor without sedative side effects following injections of low doses of propofol (15 mg/kg i.p.) [27]. We have detected EC_50_ values for modulation of the startle receptors R271Q and R271L in a tenfold to hundredfold lower concentration range. Thus, it is conceivable that 4-chloropropofol might act in sub-anesthetic doses at startle disease glycine receptors.

## Conclusions

In summary, our results broaden the body of knowledge about approaches to restore the reduced glycinergic inhibition in startle disease. In particular, 4-chloropropofol might lead to an effective enhancement of the function of startle disease glycine receptors *in vitro*. Based on our results we hypothesize that 4-chloropropofol might alleviate hyperekplexic symptoms in animal models of startle disease.

## Competing interests

The authors declare that they have no competing interest.

## Authors’ contributions

JR, JA, GH and ML participated in the design and discussion of the study. JR carried out the experimental work. Analysis and interpretation was done by JR and JA. VB and KK contributed to the methodological analysis and scientific interpretation of the data. JR, JA and ML wrote the manuscript. RD enabled the performance of the concept of the study and the realization of the experimental work in the lab of neurology. All others read and approved the final manuscript.

## Pre-publication history

The pre-publication history for this paper can be accessed here:

http://www.biomedcentral.com/1471-2377/12/104/prepub

## Supplementary Material

Additional file 1 **Osmolarity controls.** Whole cell experiments at α1R271Q- glycine receptors lack activation following 1100 mM glucose application. High glycine solutions of 300 mM glycine adjusted to pH 7.4 by Na-OH revealed 1096 mOsm. 300 mM glycine osmolarity subtracted from the osmolarity of the buffer solution itself, resulted in a total of Δ 800 mOsmol. Switch to 1100 mM glucose rather reduces baseline leak currents following 2 s application. In addition wild type glycine receptors didn’t show sensitivity for osmolarity controls (600 mM sucrose). Thus osmolarity effects resulting from high glycine concentrations up to 300 mM at startle glycine receptor mutations can be excluded. Click here for file

Additional file 2 **Control of rundown effects of glycine receptors.** Current amplitude of glycine control (10 mM) traces applied before and after application of 4-chloropropofol at α1R271K-mutation remains almost unchanged with negligible run down in liquid filament switch technique. Same picture for subsequent application of subsaturating glycine solution (10 μM) before and after co-application of 17.15 mM ethanol in custom-designed gravity driven perfusion system. There is almost no change in amplitude size. In any case the addition of ATP to the pipette solution should help to reduce receptor desensibilisation to exclude rundown effects for future studies. Click here for file

Additional file 3 **Ethanol control experiments.** Ethanol (17.15 mM) in a sub-saturating glycine solution (10 μM) doesn’t lead to an additional activation of wild type glycine receptors, by contrast the initial sub-saturating glycine response is reduced when ethanol is added. Consequently the ethanol effect in particular at high 4-chloropropofol doses (where the concentration of the diluent EtOH corresponding to the highest drug concentration is 17.15 mM) has no influence on 4-chloropropofol effect. Click here for file

Additional file 4 **Glycine sensitivity at R271Q.** α1R271Q-mutation lacks activation by low glycine concentrations (10-100 μM). Dose response curve starts with current peaks of less than 50 pA at 1 mM glycine. These current traces are depicted as example to illustrate repressed glycine sensitivity of mutated startle receptors. Click here for file
