# Supplementary material for: 4-Chloropropofol enhances chloride currents in human hyperekplexic and artificial mutated glycine receptors
Source: BMC Neurol. 2012 Sep 24;12:104. doi: 10.1186/1471-2377-12-104 (PMC3517478; doi:10.1186/1471-2377-12-104)
Supplement: Additional file 1 — Osmolarity controls. Whole cell experiments at α1R271Q- glycine receptors lack activation following 1100 mM glucose application. High glycine solutions of 300 mM glycine adjusted to pH 7.4 by Na-OH revealed 1096 mOsm. 300 mM glycine osmolarity subtracted from the osmolarity of the buffer solution itself, resulted in a total of Δ 800 mOsmol. Switch to 1100 mM glucose rather reduces baseline leak currents following 2 s application. In addition wild type glycine receptors didn’t show sensitivity for osmolarity controls (600 mM sucrose). Thus osmolarity effects resulting from high glycine concentrations up to 300 mM at startle glycine receptor mutations can be excluded. [file 1471-2377-12-104-S1.ppt]

## Slide 1
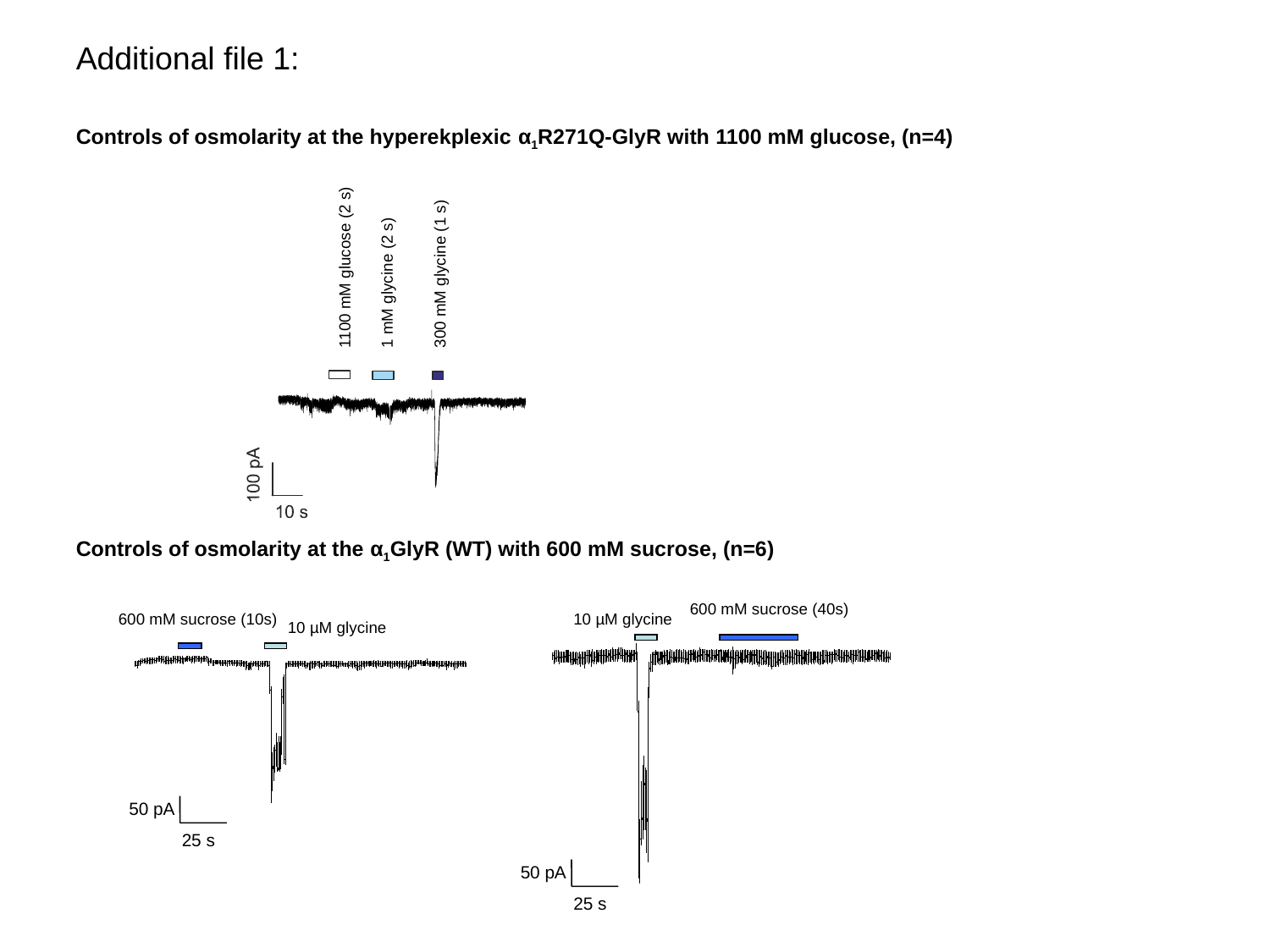

Additional file 1:
Controls of osmolarity at the hyperekplexic α1R271Q-GlyR with 1100 mM glucose, (n=4)
1100 mM glucose (2 s)
1 mM glycine (2 s)
300 mM glycine (1 s)
Controls of osmolarity at the α1GlyR (WT) with 600 mM sucrose, (n=6)
600 mM sucrose (40s)
10 µM glycine
50 pA
25 s
600 mM sucrose (10s)
10 µM glycine
50 pA
25 s
