# Supplementary material for: 4-Chloropropofol enhances chloride currents in human hyperekplexic and artificial mutated glycine receptors
Source: BMC Neurol. 2012 Sep 24;12:104. doi: 10.1186/1471-2377-12-104 (PMC3517478; doi:10.1186/1471-2377-12-104)
Supplement: Additional file 2 — Control of rundown effects of glycine receptors. Current amplitude of glycine control (10 mM) traces applied before and after application of 4-chloropropofol at α1R271K-mutation remains almost unchanged with negligible run down in liquid filament switch technique. Same picture for subsequent application of subsaturating glycine solution (10 μM) before and after co-application of 17.15 mM ethanol in custom-designed gravity driven perfusion system. There is almost no change in amplitude size. In any case the addition of ATP to the pipette solution should help to reduce receptor desensibilisation to exclude rundown effects for future studies. [file 1471-2377-12-104-S2.ppt]

## Slide 1
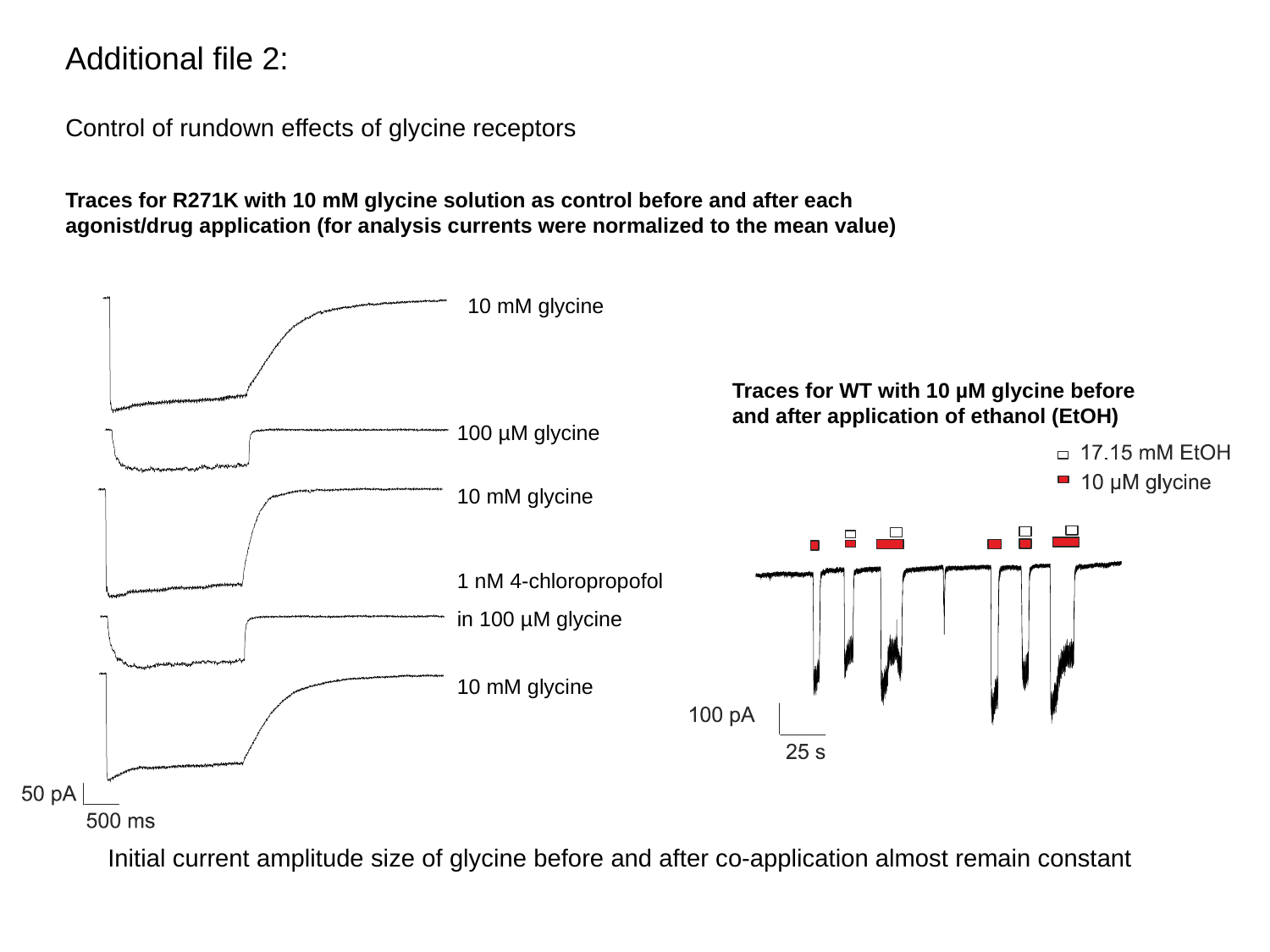

Additional file 2:
Control of rundown effects of glycine receptors
Traces for R271K with 10 mM glycine solution as control before and after each agonist/drug application (for analysis currents were normalized to the mean value)
10 mM glycine
100 µM glycine
10 mM glycine
10 mM glycine
Traces for WT with 10 µM glycine before and after application of ethanol (EtOH)
1 nM 4-chloropropofol
in 100 µM glycine
Initial current amplitude size of glycine before and after co-application almost remain constant
