# Supplementary material for: 4-Chloropropofol enhances chloride currents in human hyperekplexic and artificial mutated glycine receptors
Source: BMC Neurol. 2012 Sep 24;12:104. doi: 10.1186/1471-2377-12-104 (PMC3517478; doi:10.1186/1471-2377-12-104)
Supplement: Additional file 3 — Ethanol control experiments. Ethanol (17.15 mM) in a sub-saturating glycine solution (10 μM) doesn’t lead to an additional activation of wild type glycine receptors, by contrast the initial sub-saturating glycine response is reduced when ethanol is added. Consequently the ethanol effect in particular at high 4-chloropropofol doses (where the concentration of the diluent EtOH corresponding to the highest drug concentration is 17.15 mM) has no influence on 4-chloropropofol effect. [file 1471-2377-12-104-S3.ppt]

## Slide 1
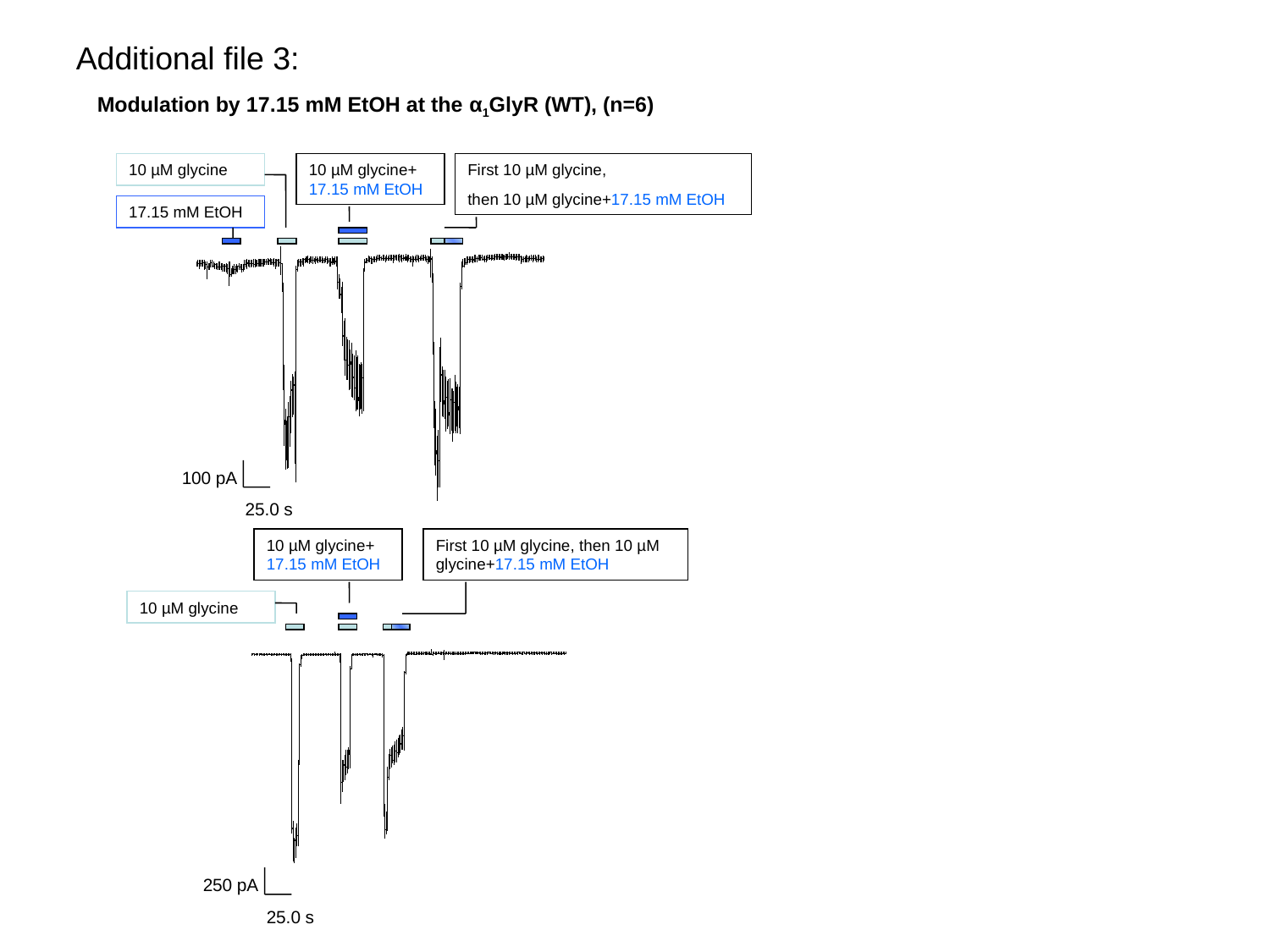

Additional file 3:
Modulation by 17.15 mM EtOH at the α1GlyR (WT), (n=6)
10 µM glycine
10 µM glycine+ 17.15 mM EtOH
First 10 µM glycine,
then 10 µM glycine+17.15 mM EtOH
17.15 mM EtOH
100 pA
25.0 s
10 µM glycine+ 17.15 mM EtOH
First 10 µM glycine, then 10 µM glycine+17.15 mM EtOH
10 µM glycine
250 pA
25.0 s
