# Supplementary material for: 4-Chloropropofol enhances chloride currents in human hyperekplexic and artificial mutated glycine receptors
Source: BMC Neurol. 2012 Sep 24;12:104. doi: 10.1186/1471-2377-12-104 (PMC3517478; doi:10.1186/1471-2377-12-104)
Supplement: Additional file 4 — Glycine sensitivity at R271Q. α1R271Q-mutation lacks activation by low glycine concentrations (10-100 μM). Dose response curve starts with current peaks of less than 50 pA at 1 mM glycine. These current traces are depicted as example to illustrate repressed glycine sensitivity of mutated startle receptors. [file 1471-2377-12-104-S4.ppt]

## Slide 1
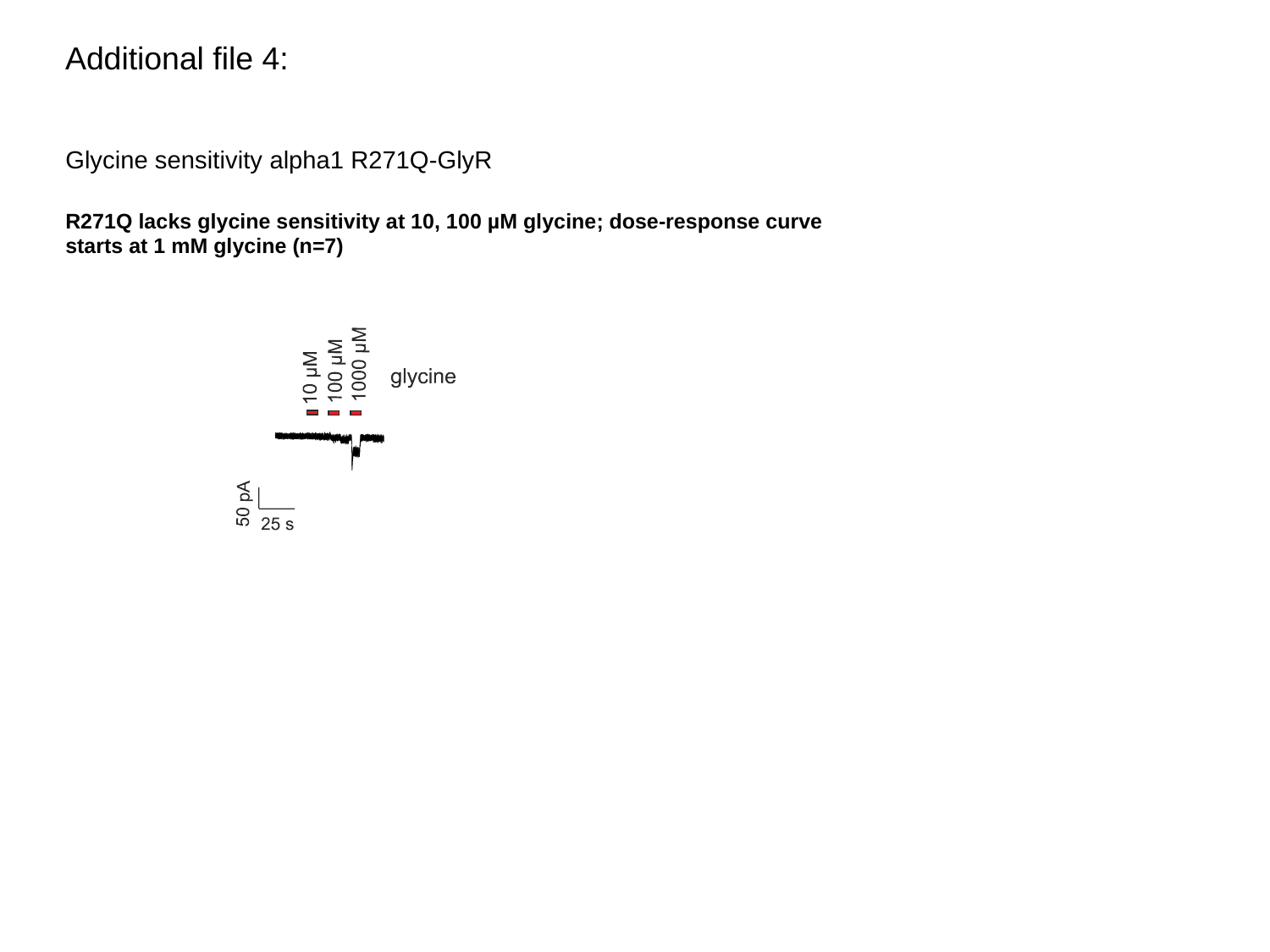

Additional file 4:
Glycine sensitivity alpha1 R271Q-GlyR
R271Q lacks glycine sensitivity at 10, 100 µM glycine; dose-response curve starts at 1 mM glycine (n=7)
